# Supplementary material for: Venomix: a simple bioinformatic pipeline for identifying and characterizing toxin gene candidates from transcriptomic data
Source: PeerJ. 2018 Jul 31;6:e5361. doi: 10.7717/peerj.5361 (PMC6074769; doi:10.7717/peerj.5361)
Supplement: Supplemental Information 6 [file peerj-06-5361-s006.gz › FinalOutput_1E-6/Acetylcholinesterase-1_1/finaltree.pdf]

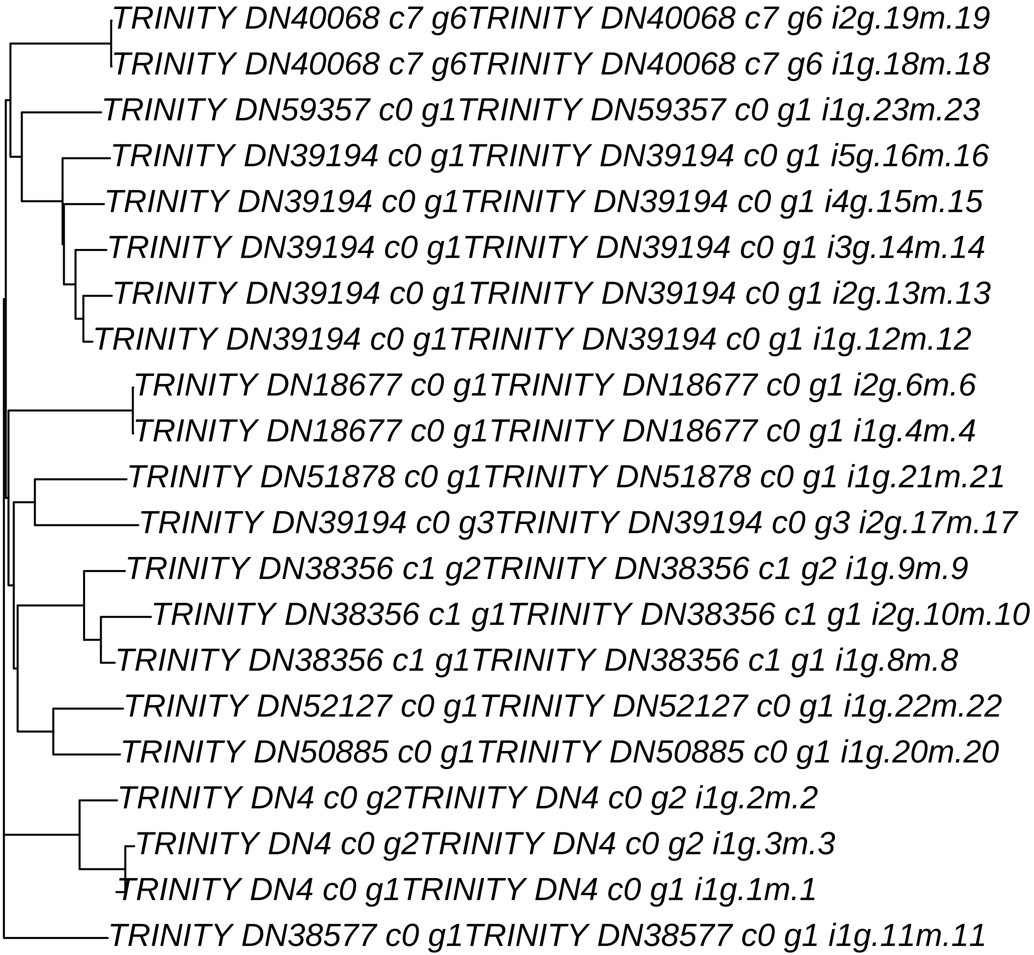
TRINITY DN40068 c7 g6TRINITY DN40068 c7 g6 i2g.19m.19
TRINITY DN40068 c7 g6TRINITY DN40068 c7 g6 i1g.18m.18
TRINITY DN59357 c0 g1TRINITY DN59357 c0 g1 i1g.23m.23
TRINITY DN39194 c0 g1TRINITY DN39194 c0 g1 i5g.16m.16
TRINITY DN39194 c0 g1TRINITY DN39194 c0 g1 i4g.15m.15
TRINITY DN39194 c0 g1TRINITY DN39194 c0 g1 i3g.14m.14
TRINITY DN39194 c0 g1TRINITY DN39194 c0 g1 i2g.13m.13
TRINITY DN39194 c0 g1TRINITY DN39194 c0 g1 i1g.12m.12
TRINITY DN18677 c0 g1TRINITY DN18677 c0 g1 i2g.6m.6
TRINITY DN18677 c0 g1TRINITY DN18677 c0 g1 i1g.4m.4
TRINITY DN51878 c0 g1TRINITY DN51878 c0 g1 i1g.21m.21
TRINITY DN39194 c0 g3TRINITY DN39194 c0 g3 i2g.17m.17
TRINITY DN38356 c1 g2TRINITY DN38356 c1 g2 i1g.9m.9
TRINITY DN38356 c1 g1TRINITY DN38356 c1 g1 i2g.10m.10
TRINITY DN38356 c1 g1TRINITY DN38356 c1 g1 i1g.8m.8
TRINITY DN52127 c0 g1TRINITY DN52127 c0 g1 i1g.22m.22
TRINITY DN50885 c0 g1TRINITY DN50885 c0 g1 i1g.20m.20
TRINITY DN4 c0 g2TRINITY DN4 c0 g2 i1g.2m.2
TRINITY DN4 c0 g2TRINITY DN4 c0 g2 i1g.3m.3
TRINITY DN4 c0 g1TRINITY DN4 c0 g1 i1g.1m.1
TRINITY DN38577 c0 g1TRINITY DN38577 c0 g1 i1g.11m.11
